# Recognition and management of rapid-onset gluten ataxias: case series

**DOI:** 10.1186/s40673-021-00139-z

**Published:** 2021-06-13

**Authors:** Laurence Newrick, Nigel Hoggard, Marios Hadjivassiliou

**Affiliations:** 1 Academic department of neurosciences, Sheffield Teaching Hospitals NHS Foundation Trust, Sheffield, UK; 2 Academic Department of Neuroradiology, Royal Hallamshire Hospital, Sheffield Teaching Hospitals NHS Foundation Trust, Sheffield, S10 2JF UK

**Keywords:** Ataxia, Cerebellar ataxia, Gluten Ataxia, Gluten sensitivity, Immune-mediated cerebellar ataxias, Cerebellitis

## Abstract

**Background:**

Most immune-mediated cerebellar ataxias, including those associated with gluten sensitivity (Gluten Ataxia), tend to present subacutely and usually progress gradually. Acute presentations with rapid progression outside the context of paraneoplastic cerebellar degeneration require prompt diagnosis and early access to disease-modifying immunotherapy in order to avert severe and permanent neurological disability.

**Case presentations:**

We describe three cases of rapid-onset Gluten Ataxia, an immune-mediated cerebellar ataxia due to gluten sensitivity. We detail their presentation, clinical and neuroimaging findings, and our treatment strategy with immunotherapy.

**Conclusions:**

Our cases highlight the potential for immune-mediated cerebellar ataxias to present acutely, with rapid-onset symptoms and devastating neurological consequences. We caution against the diagnosis of ‘post-infective cerebellitis’ in adults, and advocate early consideration of an immune-mediated cerebellar ataxia and initiation of immunotherapy to prevent irreversible cerebellar damage.

## Introduction

Immune-mediated cerebellar ataxias (IMCAs) are a heterogeneous group of disorders characterised by subacute onset, prominence of a ‘midline’ cerebellar syndrome with vermian involvement on imaging, and evidence of organ-specific autoimmunity with frequent co-occurrence of other autoimmune diseases [1–3]. Symptoms tend to manifest in middle-age, with an insidious onset and gradual progression [1]. Rarely neurological deterioration can be rapid and devastating, mimicking acute ‘post-infective’ cerebellitis or paraneoplastic cerebellar degeneration [3, 4]. Clinical features overlapping with hyperexcitability disorders in the stiff person syndrome-spectrum appear to be more common in ‘fulminant’ IMCA presentations [3]. In these acute cases, the diagnosis may be delayed by lack of awareness, early clinical overlap with the more benign ‘post-infective’ cerebellitis, and poor access to specialist ataxia centres with advanced neuroimaging techniques sensitive to early vermis dysfunction (prior to the development of atrophy) [5]. Anecdotally, our experience is that many *adult* onset ‘post-infective’ cerebellitis diagnoses are speculative, and are more likely to represent an acute presentation of an IMCA, with ultimate progression and neurological sequela [6]. Evidence supporting the potential of disease-modifying immunotherapy underlines the importance of early identification to prevent irreversible neurological disability [5, 7–9]. We describe three cases of rapid-onset Gluten Ataxia (GA), a form of IMCA due to gluten sensitivity. The cases highlight the potential for an aggressive natural history and supporting an equally aggressive approach with early immunotherapy to prevent permanent disability.

## Case presentations

### Case 1

A 34-year-old man was referred to the Sheffield Ataxia Centre with a 12 month history of progressive slurred speech, unsteadiness, intractable vomiting, weight loss and deteriorating mobility. He had initially presented with rapid-onset unsteadiness, ear pain, headache and vomiting over 2 weeks, which was attributed to a ‘viral infection’. After an initial plateau in his symptoms, he experienced relentless progression and became dependent on a four wheeled frame and wheelchair within 12 months of symptom onset. There was a past medical history of rosacea and previous moderate alcohol use. There was a family history of motor neuron disease (two maternal aunts, a maternal grandmother and a great uncle). Physical examination demonstrated a predominant cerebellar ataxia with additional mild pyramidal signs. There was severe dysarthria, marked gaze-evoked horizontal nystagmus and down-beat nystagmus on upgaze. There was no opthalmoparesis. There was severe upper and lower limb dysmetria and truncal ataxia. There was evidence of lower limb spasticity with mildly increased tone and asymmetrically brisk reflexes (left > right), in the absence of clonus and with plantar-flexor responses. Sensory testing was normal for all modalities. Gait was severely ataxic. Routine, micronutrient, vasculitic and immunological blood tests were normal (FBC, U&Es, LFTs, coagulation studies, ESR, CRP, B12, folate, TFTs, copper, zinc, selenium, ANA, ENA, complement C3/C4, c-ANCA, p-ANCA, RF, SPE, immunoglobulins, lupus anticoagulant panel, lyme serology). Serum autoimmune (anti-GAD, anti-TPO) and paraneoplastic antibody testing was unremarkable, including onconeuronal anti-cerebellar antibodies (Anti-Yo, Anti-Hu/Ri, Amphyiphysin, Anti-CRMP-5, Anti-MA2/Ta, Anti-Tr) and intracellular antibodies (anti-LGI1, anti-CASPR2, antimGluR1, anti-mGluR5, anti-NMDAR, anti-AMPAR and anti-GABA(B)). Cerebrospinal fluid (CSF) demonstrated evidence of mild inflammation with a mild pleocytosis and CSF-restricted positive IgG oligoclonal bands (WCC 5, protein 0.38, glucose 3.4 (serum 5.3), lactate 1.5). Viral PCR was negative and there were no malignant cells on CSF cytology. CSF RT-QuiC was negative. Magnetic resonance imaging (MRI) of the head and cervical spine and magnetic resonance spectroscopy (MRS) demonstrated marked vermis spectroscopic abnormalities (superior vermis N-acetylaspartate/Creatine ratio (NAA/Cr) was severely reduced at 0.54 (normal over 1), right cerebellar hemisphere ratio 0.63) with mild cerebellar vermian and hemispheric atrophy. No malignancy was identified on extensive imaging and endoscopy, including two 18F-fluoro-2-deoxy-D-glucose positron emission tomography scans (18FDG-PET). Electromyography (EMG), nerve conductions studies and electroencephalogram (EEG) were normal. Muscle biopsy, mitochondrial respiratory chain analysis and common mitochondrial mutations were normal. He had an elevated IgG anti-gliadin antibody (3.6 U/ml, abnormal > 3) and was positive for HLA-DQ2 (DQB1*02). There was no evidence of enteropathy on duodenal biopsy. He was diagnosed with GA and started treatment with prednisolone (20 mg once daily), a course of IVIG and mycophenolate (uptitrated to 1 g twice daily), approximately 10 months after symptom onset, resulting in a gradual and sustained clinical improvement. He was also started on a gluten free diet at the same time. Two years into treatment he was able to walk with the assistance of one, mirrored by improvements in MRI spectroscopy (at 2 years, vermian NAA/Cr ratio 0.7). He has remained stable over 5 years of follow up.

### Case 2

**Fig. 1:**
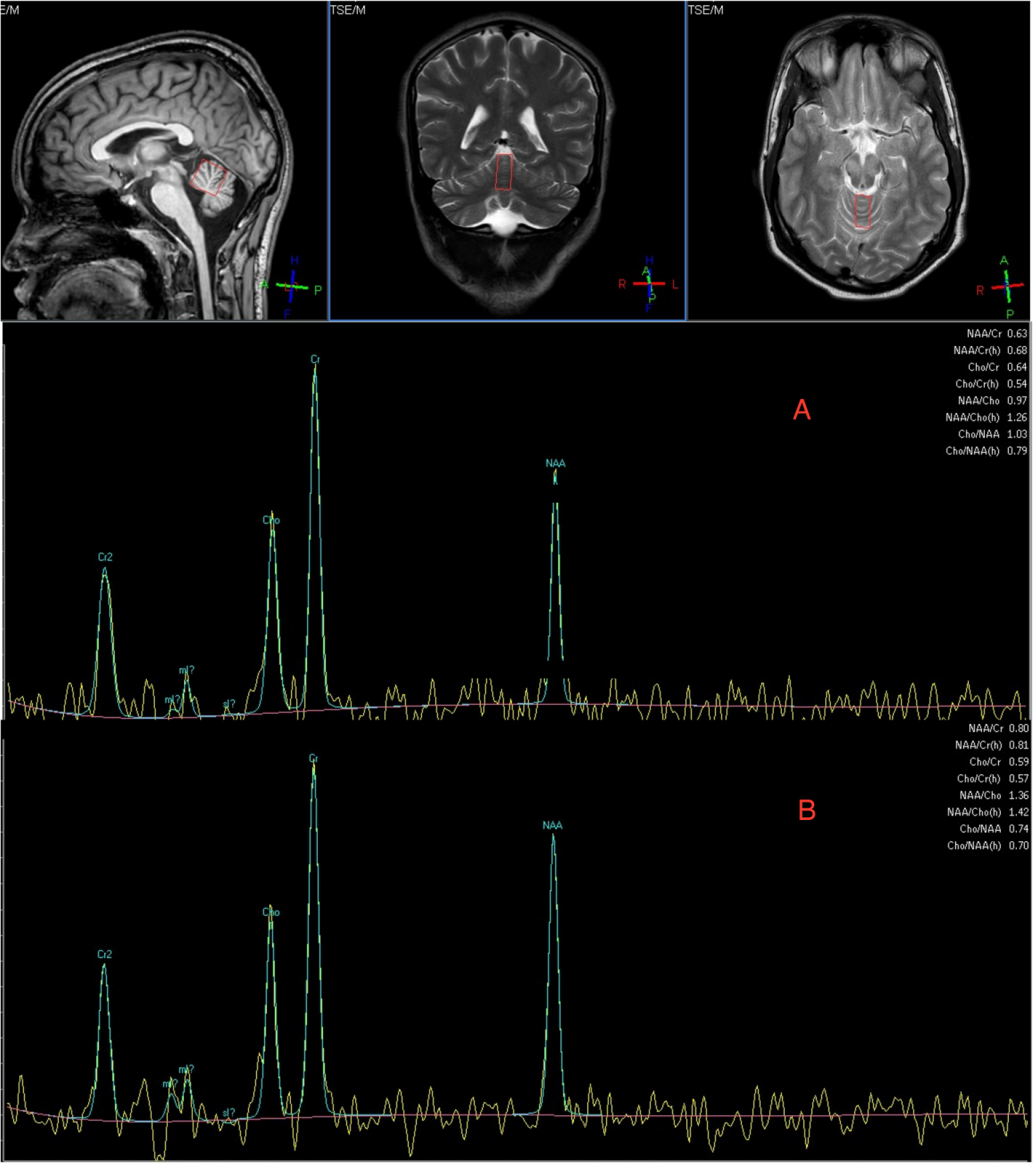
Magnetic resonance spectroscopy (MRS) of case 2 pre-treatment (**A**) and after 1 year of immunotherapy and gluten free diet (**B**). Pre-treatment there is disproportionate vermian spectroscopic abnormalities of the N-acetyl aspartate/Creatine ratio (NAA/Cr) from the vermis (0.63, normal > 1) in the presence of only mild atrophy. MR spectroscopy is a sensitive marker of cerebellar dysfunction prior to the development of atrophy and correlates with clinical severity. Following treatment, there has been significant improvement in vermian spectroscopic abnormalities with NAA/Cr ratio 0.80 in parallel with clinical improvement. MR spectroscopy is a sensitive marker for monitoring disease progression and treatment response which is not possible with just volumetric imaging. The patient has made almost a full recovery with just very minor tandem walking difficulties

A 19-year-old man was referred to the Sheffield Ataxia Centre with a 12-month history of progressive slurred speech, difficulties swallowing, incoordination, imbalance and fluctuating abdominal pain and diarrhoea. Past medical history was remarkable for bacterial meningitis age 6 months. There was no significant family history. There was a history of ketamine, alprazolam and nitrous oxide misuse. At the time of referral he was using thickened fluids and required the assistance of one and a frame to mobilise. Physical examination demonstrated a cerebellar syndrome with pyramidal signs and pseudobulbar affect. There was broken pursuit and slight restriction of right lateral gaze, without nystagmus. Speech was dysarthric with slowed palatal and tongue movements. He was only able to say yes or no. There was generalised limb dysmetria. There was asymmetrical increased tone in the left upper and lower limbs with generalised brisk reflexes and plantar-flexor responses. Sensory testing for all modalities was normal. Gait was spastic and ataxic. A metabolic screen (including Wilson’s disease) and micronutrients were normal. Autoimmune (including anti-AQP4 and anti-MOG) and paraneoplastic antibodies were negative. IgA anti-transglutaminase type 6 (TG6) antibodies were strongly positive (11.1 U/ml, normal range < 4), suggestive of gluten sensitivity prone to neurological dysfunction. An initial lumbar puncture demonstrated an acellular CSF and CSF-restricted oligoclonal bands. An MRI head and MRS demonstrated mild cerebellar vermian and hemispheric atrophy with spectroscopic abnormalities (NAA/Cr superior vermis 0.63 and right cerebellar hemisphere 0.67) (Fig. 1 A). There were significant brainstem changes with signal change affecting the midbrain and extending into the thalamus and corticospinal tracts bilaterally, with no enhancement. 18FDG-PET demonstrated a region of increased uptake in the small bowel. Small bowel enteroscopy was macroscopically and microscopically normal, with no evidence of giardia, T Whipplei, acid fast bacillus or malignancy. Serum Cholestanol was normal. Muscle biopsy mitochondrial respiratory chain function, mitochondrial genetic analysis (MERRF, MELAS or NARP/MILS) and genetic testing for expansion mutations (Friedreich ataxia, Dentatorubral pallidoluysian atrophy and Spinocerebellar ataxia 1, 2, 3, 6, 7, 12 and 17) were unremarkable. EMG demonstrated an abnormal blink response (failure of second R2 response when undertaking blink reflex studies) suggesting a degree of cortical hyperexcitability. There were no other features of a peripheral neuropathy or continuous limb/paraspinal motor unit activity to suggest stiff person syndrome. Prior to referral, there was no observed response to an initial tapering course of steroids and azathioprine, which was stopped. He was diagnosed with GA and started a gluten free diet and mycophenolate (uptitrated to 1 g twice daily) following an induction course of IVIG, approximately 14 months after symptom onset. He was discharged to a neurorehabilitation unit. There was evidence of spectroscopic improvement on an MRI spectroscopy performed 3 months after treatment (NAA/Cr vermis 0.79 and right cerebellar 0.85) with persistent cerebellar atrophy and diencephalic signal changes (Fig. 1 B). Over 3 years of follow up there has been a dramatic and sustained improvement in speech and mobility. He is now able to have a normal conversation with minimal dysarthria and has started running again. There is evidence of a very subtle residual gait ataxia on independent tandem walking only.

### Case 3

**Fig. 2:**
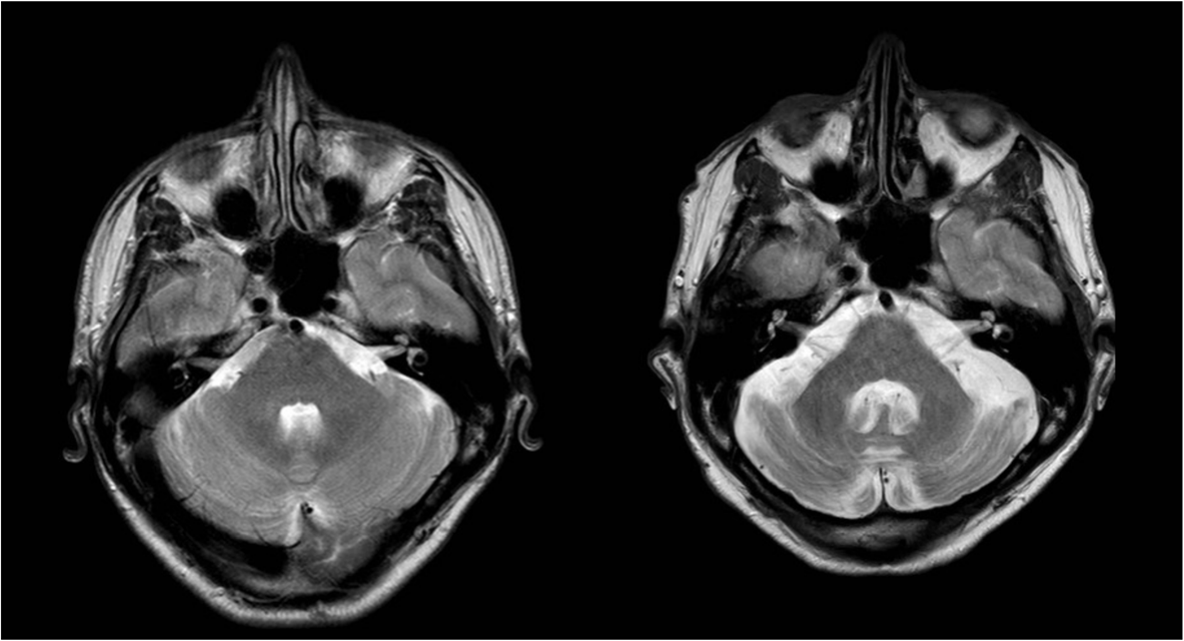
MRI (patient 3) at presentation (left) and 2 years later (right) showing the rapid development of cerebellar atrophy as a result of delayed diagnosis and treatment with gluten free diet and immunosupression

A 48-year-old man was referred to the Sheffield Ataxia Centre following rapid onset of double vision, slurred speech and imbalance 2 years prior. His symptoms had remained static but had resulted in him becoming wheelchair bound within a few weeks of onset. On presentation he was diagnosed with viral cerebellitis and treated with IV methylprednisolone, aciclovir, and a later slow weaning dose of oral steroids, with minimal improvement in symptoms. He received no further immunosuppression. Initial MRI imaging demonstrated cerebellar signal change suggestive of cerebellitis with later progressive cerebellar atrophy on interval scans (Fig. 2 A and B). He had been discharged to a neurorehabilitation unit. Past medical history was remarkable for coeliac disease diagnosed with duodenal biopsy 6 months prior to the neurological presentation. Family history was significant for a mother with dermatitis herpetiformis and a brother with coeliac disease. Prior to the referral, he had started a gluten free diet. On referral, physical examination demonstrated a cerebellar syndrome with gaze-evoked nystagmus, downbeat nystagmus in primary gaze, hypometric saccades, broken pursuit, severe dysarthria, severe limb dysmetria with mild asymmetry (left > right) and gait ataxia. He required the assistance of two people to transfer. There was evidence of myoclonus, prominent in the left upper limb, but not stimulus sensitive. As he was already established on a strict gluten free diet, all antigliadin antibodies were negative at time of referral. Autoimmune and paraneoplastic antibodies, including GAD, onconeuronal, anti-GlyR and IgG anti-DPPX were negative. A lumbar puncture performed at presentation demonstrated an acellular CSF (WCC 2, protein 0.55, negative oligoclonal bands). MRI head and MRS performed on referral (2 years into the illness), demonstrated established cerebellar vermian and hemispheric atrophy (Fig. 2 B) with spectroscopic abnormalities (superior vermis NAA/Cr 0.7, right hemisphere 0.89). Duodenal biopsy, performed after 12 months of a gluten free diet, demonstrated increased intraepithelial lymphocytes consistent with a gluten sensitive enteropathy (Marsh grade 1) but without evidence of refractory coeliac disease. Somatosensory evoked potentials (SSEP) and EEG/EMG polygraphy demonstrated no evidence of cortical hyperexcitability, cortically-driven myoclonus or stimulus-sensitive myoclonus. A diagnosis of GA was made and a tapering dose of steroids was continued. In the presence of static symptoms and an antibody/endoscopic response to a gluten free diet with stable MRS, no further immunosuppression was pursued. Mobility, however has remained stable during a period of follow up over 5 years. MR spectroscopy has remained stable.

## Discussion

The cerebellum appears particularly susceptible to autoimmunity [1, 2]. Emerging evidence suggests that a significant proportion of idiopathic sporadic cerebellar ataxias may in fact represent immune-mediated disorders, with implications for early identification and immunotherapy [10]. While paraneoplastic cerebellar degeneration and GA are well characterised IMCAs with a known trigger, for many a specific antigenic trigger or pathogenic antibody has yet to be identified [3]. In these cases, an immune aetiology is inferred by the presence of markers of autoimmunity (such as non-pathogenic autoantibodies, CSF-restricted oligoclonal bands or pleocytosis), co-occurrence or family history of autoimmune disease, and absence of a family history suggesting a genetic ataxia [3]. Associated antibodies may represent an autoimmune epiphenomena rather than be pathogenic [1]. In contrast to the ‘global’ cerebellar dysfunction of genetic and degenerative ataxias, IMCAs are characterised by a ‘midline’ cerebellar phenotype with gait ataxia disproportionate to limb, speech and ocular manifestations [1]. Neuroimaging supports a predilection for the vermis, with evidence of selective MR spectroscopic changes (NAA/creatine ratio) corresponding to clinical severity, even prior to the emergence of atrophy [5]. Symptoms tends to occur in middle-age, with insidious onset and gradual progression [1].

Rarely neurological deterioration in the context of IMCA can be rapid and devastating, even in the absence of a paraneoplastic process [7]. Acute IMCA presentations can be initially misdiagnosed as a ‘post-infective cerebellitis’, a monophasic primarily paediatric syndrome which is poorly defined and rare in adults [6]. In many cases, evidence of an infective aetiology is speculative and not supported by serological testing [6]. Our experience is that a significant proportion of such cases represent the first presentation of an IMCA and may ultimately progress without treatment, rather than follow a monophasic course [4, 6]. All three of our cases were diagnosed as possible ‘post-infective cerebellitis’ at initial presentation.

All of the patients described here had serological evidence of gluten sensitivity and one also had enteropathy in keeping with coeliac disease. We have previously described a large cohort of patients with GA that respond well to a gluten free diet alone [5, 11]. The novelty of this report is the acute presentation and rapid deterioration. In our experience a gluten free diet alone takes several months to be effective, given that the elimination of gluten sensitivity related antibodies can take up to 6 months provided strict adherence to the diet [11]. Given the rapidity of progression such cases as described here require immediate treatment with immunotherapy to prevent further cerebellar insult whilst the gluten free diet starts to have an effect. Indeed patient 2 did extremely well with impressive recovery because he was treated early (at presentation) with steroids, prior to being referred to the Sheffield Ataxia Centre. A growing base of retrospective and observational evidence supports the use of various immunotherapy treatments in the management of IMCAs, in additional to removal of any antigenic stimulus [9]. These include steroids, intravenous immunoglobulins, mycophenolate and rituximab [1, 7–9]. Regardless of modality, clinically meaningful outcomes appear to be dependent on instigating treatment early, to stop cerebellar cell loss beyond an irreversible threshold (sometimes referred to as cerebellar reserve) [9]. In rapidly progressive cases the use of steroids and intravenous immunoglobulins to provide a rapid induction prior to mycophenolate and then gluten free diet reaching full efficacy is advisable [8]. In our described cases, clinical stability or improvement was achieved only with aggressive immunotherapy.

Evidence of CNS hyperexcitability such as cortical myoclonus, abnormal blink response and exaggerated startle, is a feature of immune ataxias [1]. Evidence of such an association is strongest in anti-GAD related diseases and ataxia associated with refractory coeliac disease [1, 12]. One of our cases developed myoclonus, one demonstrated an abnormal blink response, and two had evidence of increased truncal or limb tone with pathologically brisk reflexes.

Our cases highlight the rare potential for GA to present acutely, with rapid-onset symptoms and devastating neurological consequences. We caution against the diagnosis of ‘post-infective cerebellitis’ in adults, and advocate early consideration of an IMCAs and initiation of immunotherapy in addition to gluten free diet to prevent irreversible cerebellar damage.

## Data Availability

The datasets generated and/or analysed during the current study are not publicly available as they would compromise the anonymity of the patients involved but specific data elements may be available from the corresponding author on reasonable request.

## Abbreviations

AMPAR: Alpha-amino-3-hydroxy-5-methyl-4-isoxazolepropionic acid receptor
ANA: Antinuclear antibodies
AQP4: Aquaporin 4 antibody
B12: Vitamin B12
CASPR2: Contactin-associated protein-like 2
CRP: C-reactive protein
CSF: cerebrospinal fluid
c-ANCA: Cytoplasmic antineutrophil cytoplasmic antibodies
EEG: Electroencephalogram
EMG: Electromyography
ENA: Extractable nuclear antigens
ESR: Erythrocyte sedimentation rate
FBC: Full blood count
18FDG-PET: 18F-fluoro-2-deoxy-D-glucose positron emission tomography
GA: Gluten Ataxia
GABA(B): Gamma-aminobutyric acid B
GAD: Glutamic acid decarboxylase autoantibodies
GlyR: Glycine receptor antibody
IgG: Immunoglobulin G
IgA t-TG 6: Immunoglobulin A anti-tissue transglutaminase type 6 antibodies
IgG anti-DPPX: Immunoglobulin G anti-dipeptidyl-peptidase-like protein 6 antibodies
IMCA: Immune mediated cerebellar ataxia
LFTs: Liver function tests
LGI1: Leucine-rich, glioam inactivated 1 antibody
MELAS: Mitochondrial Encephalopathy, Lactic Acidosis, and Stroke-like episodes
MERRF: Myoclonic epilepsy with ragged-red fibers
mGluR1: Metabotropic glutamate receptor 1
mGluR5: Metabotropic glutamate receptor 5
MOG: Myelin oligodendrocyte glycoprotein
MRI: Magnetic resonance imaging
MRS: Magnetic resonance spectroscopy
NAA/Cr: N-acetyl aspartate/Creatine ratio
NARP/MILS: Neurogenic muscle weakness, ataxia, and retinitis pigmentosa/maternally inherited Leigh syndrome
NMDAR: N-methyl D-aspartate receptor
p-ANCA: Perineuclear antineutrophil cytoplasmic antibodies
PCR: Polymerase chain reaction
RF: Rheumatoid factor
RT-QuiC: Real-time quaking-induced conversion.
SPE: Serum protein electrophoresis
TFTs: Thyroid function tests
TPO: Thyroid peroxidase antibody
U&Es: Urea and electrolytes
WCC: White cell count
